# Supplementary material for: Allelic strengths of encephalopathy-associated UBA5 variants correlate between in vivo and in vitro assays
Source: medRxiv. 2023 Jul 23:2023.07.17.23292782. Preprint. [Version 1] doi: 10.1101/2023.07.17.23292782 (PMC10371176; doi:10.1101/2023.07.17.23292782)
Supplement: Supplementary file 1 [file media-1.pdf]

Supplemental Information

Allelic strengths of encephalopathy-associated *UBA5* variants correlate between *in vivo* and *in vitro* assays

Xueyang Pan[1,2], Albert N. Alvarez[3], Mengqi Ma[1,2], Shenzhao Lu[1,2], Michael W. Crawford[3], Lauren C. Briere[4], Oguz Kanca[1,2], Shinya Yamamoto[1,2,5], David A. Sweetser[4,6], Jenny L. Wilson[7], Ruth J. Napier[3,8,9], Jonathan N. Pruneda[3,#], Hugo J. Bellen[1,2,5,#]

1 Department of Molecular and Human Genetics, Baylor College of Medicine, Houston, TX 77030, USA

2 Jan & Dan Duncan Neurological Research Institute, Texas Children's Hospital, Houston, TX 77030, USA

3 Department of Molecular Microbiology & Immunology, Oregon Health & Science University, Portland, OR 97239, USA

4 Center for Genomic Medicine, Massachusetts General Hospital, Boston, MA 02114, USA

5 Department of Neuroscience, Baylor College of Medicine, Houston, TX 77030, USA

6 Division of Medical Genetics & Metabolism, Massachusetts General Hospital for Children, Boston, MA 02114, USA

7 Division of Pediatric Neurology, Department of Pediatrics, Oregon Health & Science University, Portland, OR 97239, USA

8 VA Portland Health Care System, Portland, OR 97239, USA

9 Division of Arthritis & Rheumatic Diseases, Oregon Health & Science University, Portland, OR 97239, USA

# Correspondence: hbellen@bcm.edu, pruneda@ohsu.edu

Case report

The reported proband is a boy with axial hypotonia, generalized dystonia, lower extremity spasticity, global developmental delay, esotropia and failure to thrive. An electroencephalogram (EEG) showed multifocal epileptiform discharges in drowsiness and sleep. However, he has not had seizures. Two magnetic resonance imaging (MRI) studies at different ages were read as normal, although on review show a slightly thin corpus callosum, posterior periventricular white matter T2 hyperintensity resulting in increased conspicuity of the subcortical U-fibers and widening of the Sylvian fissures (Figure S1). He is not microcephalic. For more information about the clinical record of the proband please contact the corresponding author.

On trio exome sequencing, the proband was found to have compound heterozygous variants in *UBA5*, NM_024818.6:c.169A>G (p.Met57Val) and c.935A>T (p.Gln312Leu). The two variants are in *trans* phase. The p.Met57Val variant has been reported in one individual with DEE44 (Colin *et al*, 2016). The p.Gln312Leu variant has not been previously reported. Neither variant is reported in the gnomAD database v.2.1.1 (https://gnomad.broadinstitute.org). Both variants are predicted to be damaging or probably damaging by multiple pathogenicity prediction tools (Table S3).

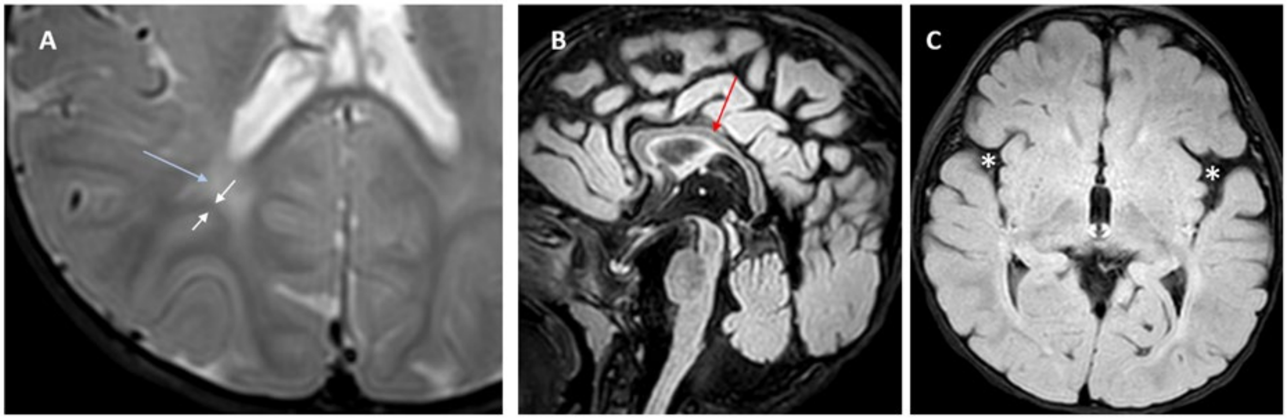

Figure S1. Brain magnetic resonance imaging (MRI) images

(A) Axial T2 image showing periventricular T2 hyperintensity (blue arrow) resulting in prominence of the subcortical U-fibers (white arrows).

(B) Sagittal flair showing mild thinning of the corpus callosum (red arrow).

(C) Axial flair image demonstrating widening of the sylvian fissures (white asterisks).

Table S1. Summary of genotypes of the reported cases

References	Family	Allele #1	Allele #2
Colin, *et al.*, 2016 (Colin *et al.*, 2016)	A	p.Ala371Thr (IA[†])	p.Gln302*
	B	p.Ala371Thr (IA)	p.Lys324Asnfs*14
	C	p.Asp389Tyr (IA)	p.Val260Met (II)
	D	p.Met57Val (II)	p.Gly168Glu (III)
Muona, *et al.*, 2016 (Muona *et al*, 2016)	A	p.Ala371Thr (IA)	p.Arg55His (III)
	B	p.Ala371Thr (IA)	p.Tyr285*
	C, E	p.Ala371Thr (IA)	p.Arg188*
	D	p.Ala371Thr (IA)	p.Arg61*
Arnadottir, *et al.*, 2017 (Arnadottir *et al*, 2017)		p.Ala371Thr (IA)	p.Ala288= (splicing variant)
Daida, *et al.*, 2018 (Daida *et al*, 2018)		p.Tyr72Cys (IB)	Deletion
Mignon-Ravix, *et al.*, 2018 (Mignon-Ravix *et al*, 2018)		p.Tyr53Phe (II)[††]	p.Tyr53Phe (II)
Low, *et al.*, 2019 (Low *et al*, 2019)		p.Asp389Gly (IA)	Deletion
Briere, *et al.*, 2021 (Briere *et al*, 2021)	A	p.Ala371Thr (IA)	p.Cys303Arg (III)
	B	p.Ala371Thr (IA)	p.Arg188*
	C	p.Ala371Thr (IA)	p.Leu254Pro (III)
	D	p.Ala371Thr (IA)	p.Cys303Arg (III)
This study		p.Met57Val (II)	p.Gln312Leu (IB)

[†] The variant classification using fly phenotypic assays (results shown in Figure 3)

†† Consanguineous family

Table S2. Clinical features of individuals with *UBA5*-associated DEE44

(See separate Excel table)

Table S3. Bioinformatic predictions of the pathogenicity of reported *UBA5* variants

	Variant 1	Variant 2
Genomic position (GRCh38)	3:132665830A>G	3:132394214A>T
Amino acid change	p.Met57Val	p.Gln312Leu
Allele frequency in gnomAD	Absent	Absent
CADD score	25.3	29.1
SIFT	Damaging	Damaging
PolyPhen2	1.000 (probably damaging)	0.999 (probably damaging)
MutationTaster	Disease causing	Disease causing
PROVEAN	-3.18 (deleterious)	-6.59 (deleterious)

References

Arnadottir GA, Jensson BO, Marelsson SE, Sulem G, Oddsson A, Kristjansson RP, Benonisdottir S, Gudjonsson SA, Masson G, Thorisson GA *et al* (2017) Compound heterozygous mutations in UBA5 causing early-onset epileptic encephalopathy in two sisters. *BMC Med Genet* 18: 103

Briere LC, Walker MA, High FA, Cooper C, Rogers CA, Callahan CJ, Ishimura R, Ichimura Y, Caruso PA, Sharma N *et al* (2021) A description of novel variants and review of phenotypic spectrum in UBA5-related early epileptic encephalopathy. *Cold Spring Harb Mol Case Stud* 7

Colin E, Daniel J, Ziegler A, Wakim J, Scrivo A, Haack TB, Khiati S, Denomme AS, Amati-Bonneau P, Charif M *et al* (2016) Biallelic Variants in UBA5 Reveal that Disruption of the UFM1 Cascade Can Result in Early-Onset Encephalopathy. *Am J Hum Genet* 99: 695-703

Daida A, Hamano SI, Ikemoto S, Matsuura R, Nakashima M, Matsumoto N, Kato M (2018) Biallelic loss-of-function UBA5 mutations in a patient with intractable West syndrome and profound failure to thrive. *Epileptic Disord* 20: 313-318

Low KJ, Baptista J, Babiker M, Caswell R, King C, Ellard S, Scurr I (2019) Hemizygous UBA5 missense mutation unmasks recessive disorder in a patient with infantile-onset encephalopathy, acquired microcephaly, small cerebellum, movement disorder and severe neurodevelopmental delay. *Eur J Med Genet* 62: 97-102

Mignon-Ravix C, Milh M, Kaiser CS, Daniel J, Riccardi F, Cacciagli P, Nagara M, Busa T, Liebau E, Villard L (2018) Abnormal function of the UBA5 protein in a case of early developmental and epileptic encephalopathy with suppression-burst. *Hum Mutat* 39: 934-938

Muona M, Ishimura R, Laari A, Ichimura Y, Linnankivi T, Keski-Filppula R, Herva R, Rantala H, Paetau A, Poyhonen M *et al* (2016) Biallelic Variants in UBA5 Link Dysfunctional UFM1 Ubiquitin-like Modifier Pathway to Severe Infantile-Onset Encephalopathy. *Am J Hum Genet* 99: 683-694
